# Prediction of Pregnancy Complications Related to Placental Dysfunction: Protocol for a Prospective Cohort Study (Placental Health Study)

**DOI:** 10.2196/95231

**Published:** 2026-08-25

**Authors:** Luhao Han, Olivia Holland, Hasini Rathnayake, Cristiane de Freitas Paganoti, Conrado Sávio Ragazini, Elsa Suk Fan Chan, Sally Mahler, Carman Wing Sze Lai, Daniel Lorber Rolnik, Anthony Perkins, David Ellwood, Sailesh Kumar, Fabricio Da Silva Costa

**Affiliations:** 1 School of Pharmacy and Medical Sciences, Griffith University, Gold Coast, Queensland, Australia; 2 Women-Newborn-Children-Services, Gold Coast University Hospital, Gold Coast, Queensland, Australia; 3 Department of Medical Laboratory Science, Faculty of Allied Health Sciences, University of Peradeniya, Peradeniya, Sri Lanka; 4 Maternal Fetal Medicine Unit, Women-Newborn-Children Services, Gold Coast University Hospital, Gold Coast University Hospital, Level 1 B Block, B.1.096, 1 Hospital Boulevard, Gold Coast, Queensland, 4215, Australia, 61 07 5687 1149; 5 School of Medicine and Dentistry, Griffith University, Gold Coast, Queensland, Australia; 6 Department of Obstetrics and Gynaecology, Monash University, Melbourne, Victoria, Australia; 7 Women’s and Newborn, Monash Health, Melbourne, Victoria, Australia; 8 School of Health, University of the Sunshine Coast, Sunshine Coast, Australia; 9 Mater Research Institute, The University of Queensland, Brisbane, Queensland, Australia; 10 Women’s and Newborn Services, Royal Brisbane and Women’s Hospital, Brisbane, Queensland, Australia

**Keywords:** placental insufficiency, preeclampsia, fetal growth restriction, biomarker, prediction model, risk assessment, prospective studies

## Abstract

**Background:**

Placental dysfunction underlies major obstetric complications, including preeclampsia, fetal growth restriction, preterm birth, and stillbirth. While early identification of pregnancies at high risk of complications enables preventive interventions, current screening methods based on traditional risk factors have limited accuracy.

**Objective:**

This study aims to develop robust prediction models for placental dysfunction–related pregnancy disorders across 3 gestational windows and to determine the optimal delivery time by integrating maternal characteristics, biophysical measurements, and maternal circulating biomarkers.

**Methods:**

The Placental Health Study is a prospective cohort study conducted at the Maternal Fetal Medicine Unit at Gold Coast University Hospital, Australia. Singleton pregnancies are enrolled at 11 to 13 weeks, 26 to 28 weeks, and 35 to 36 weeks of gestation and are followed until delivery. Exclusion criteria are multiple pregnancies, major fetal anomalies, maternal age below 18 years, and inability to provide informed consent. Clinical data, standardized ultrasound and Doppler measurements, hemodynamic parameters, and biospecimens (serum, plasma, whole blood RNA, and urine) are collected at each visit. Three gestational age-specific datasets (11‐13 weeks, 26‐28 weeks, and 35‐36 weeks) will be constructed for model development. Prediction models will be developed using multivariable logistic regression. Competing risks (Fine and Gray) models will be used as sensitivity analyses for outcomes related to gestational age at delivery. Bayesian updating will be applied to refine existing Fetal Medicine Foundation risk estimates using additional biomarkers or variables collected later in pregnancy. Developed models will undergo internal validation via 5-fold cross-validation, with performance assessed through model discrimination, calibration, and comparison against existing approaches. Collected biospecimens will also be analyzed for angiogenic markers and exploratory omics studies.

**Results:**

Recruitment occurred from June 2022 to February 2026, enrolling 1267 participants. As of submission, 1072 (84.6%) participants have completed their pregnancies, 144 (11.4%) participants remain active in follow-up, and 51 (4%) were withdrawn or had missing or duplicated data. Clinical data have undergone preliminary cleaning and descriptive analysis. Biospecimens are being processed for biomarker quantification and exploratory molecular analyses. Two to three publications are anticipated in late 2026 or early 2027.

**Conclusions:**

This cohort establishes a comprehensive database and biospecimen repository to advance prediction of placental dysfunction–related complications. Three-stage recruitment integrated into routine clinical care enhances feasibility and translation potential, enabling dynamic risk assessment and investigation of temporal biomarker changes. Although the single-center design may limit generalizability, warranting future external validation, the interdisciplinary research team brings diverse expertise that strengthens study depth and scope.

## Introduction

Placental dysfunction represents a critical pathogenic mechanism underlying pregnancy complications, including preeclampsia, fetal growth restriction (FGR), preterm birth, and stillbirth [1,2]. These disorders collectively affect around 10% of pregnancies worldwide, significantly contributing to maternal and perinatal mortality and morbidity [3-6]. They impose a substantial health care burden through increased surveillance requirements, emergency cesarean deliveries, and neonatal intensive care [7-9], and also increase long-term cardiovascular and metabolic disease risks for both mothers and offspring [10,11].

Preeclampsia and FGR frequently coexist with evidence of impaired placentation and placental function, increasing the risks of preterm birth and stillbirth [12,13]. Research has shown that these complications share common pathogenic pathways characterized by complex maternal-fetal-placental interactions [2,3,14]. Placental dysfunction acts as the primary pathophysiological driver, originating from impaired trophoblast invasion and uteroplacental spiral artery remodeling during early pregnancy, resulting in reduced placental perfusion and insufficiency [1,15,16].

Early risk identification of high-risk pregnancies enables targeted preventive interventions and reduces unnecessary intensive care for low-risk cases. Traditional screening methods based on maternal risk factors lack precision, but recent advances in biophysical and biochemical assessments have improved early risk assessment accuracy [17]. Doppler ultrasound measurements from maternal uterine and ophthalmic arteries can provide valuable insights into endothelial health and placental insufficiency [13,18,19]. Additionally, maternal serum angiogenic factors, specifically soluble fms-like tyrosine kinase-1 (sFlt-1) and placental growth factor (PlGF) have emerged as robust biomarkers for screening and diagnosing preeclampsia and other placenta-related complications [20,21].

The first-trimester prediction model developed by the Fetal Medicine Foundation (FMF), integrating maternal factors, mean arterial pressure (MAP), uterine artery pulsatility index (UtAPI), and PlGF, identifies approximately 90% of early preeclampsia (requiring delivery <32 weeks), 75% of preterm preeclampsia (delivery <37 weeks), and 41% of term preeclampsia cases at a 10% screen-positive rate [22]. This approach also detects 56% of early small for gestational age (SGA; delivery <32 weeks) and 46% of preterm SGA (delivery <37 weeks) neonates [23]. Clinical trials have demonstrated that prophylactic aspirin (>100 mg) combined with the first-trimester prediction model effectively prevents preterm preeclampsia and its associated adverse outcomes [24].

Despite these advances, predicting and preventing late-onset or term preeclampsia and FGR remains challenging due to their heterogeneous pathophysiology. These conditions exhibit less pronounced angiogenic biomarker alterations and weaker associations with placental dysfunction than early-onset cases [25]. Although causing fewer adverse outcomes, term complications have a higher prevalence, thus constituting a substantial health care burden [26]. Furthermore, low-dose aspirin appears to be less effective in preventing term preeclampsia [24]. Recent research proposes that risk stratification at 35 to 36 weeks of gestation with planned birth may offer a potential preventive strategy for managing term preeclampsia [27]. However, determining optimal delivery timing based on individualized risk stratification requires more investigation. Emerging evidence links maternal cardiovascular maladaptation and altered hemodynamic profiles with preeclampsia and FGR development, providing new directions for improving prediction models [28,29]. Current research remains limited on combining hemodynamic measurements with placental dysfunction markers to enhance prediction accuracy.

This prospective cohort study aims to develop enhanced prediction models for placental dysfunction–related complications and define optimal delivery timing. Candidate predictors will be selected a priori based on four guiding principles: (1) established inclusion in validated prediction models and current screening approaches (maternal characteristics, MAP, uterine artery Doppler, and angiogenic biomarkers used in the FMF prediction models), (2) biological plausibility and prior evidence linking placental dysfunction and maternal cardiovascular maladaptation with preeclampsia and FGR (eg, ophthalmic artery Doppler and arterial stiffness measures), (3) feasibility of implementation within routine maternity care pathways, and (4) the potential incremental predictive value of novel biomarkers derived from stored biospecimens and exploratory omics approaches. By integrating clinical factors, biophysical parameters, and maternal circulating biomarkers across 3 critical gestational windows, this study will improve risk stratification and guide clinical decision-making in perinatal care. Furthermore, the comprehensive dataset and biobank will facilitate investigation of novel biomarkers and pathophysiological mechanisms underlying placental dysfunction.

## Methods

### Study Design

**Figure 1.**
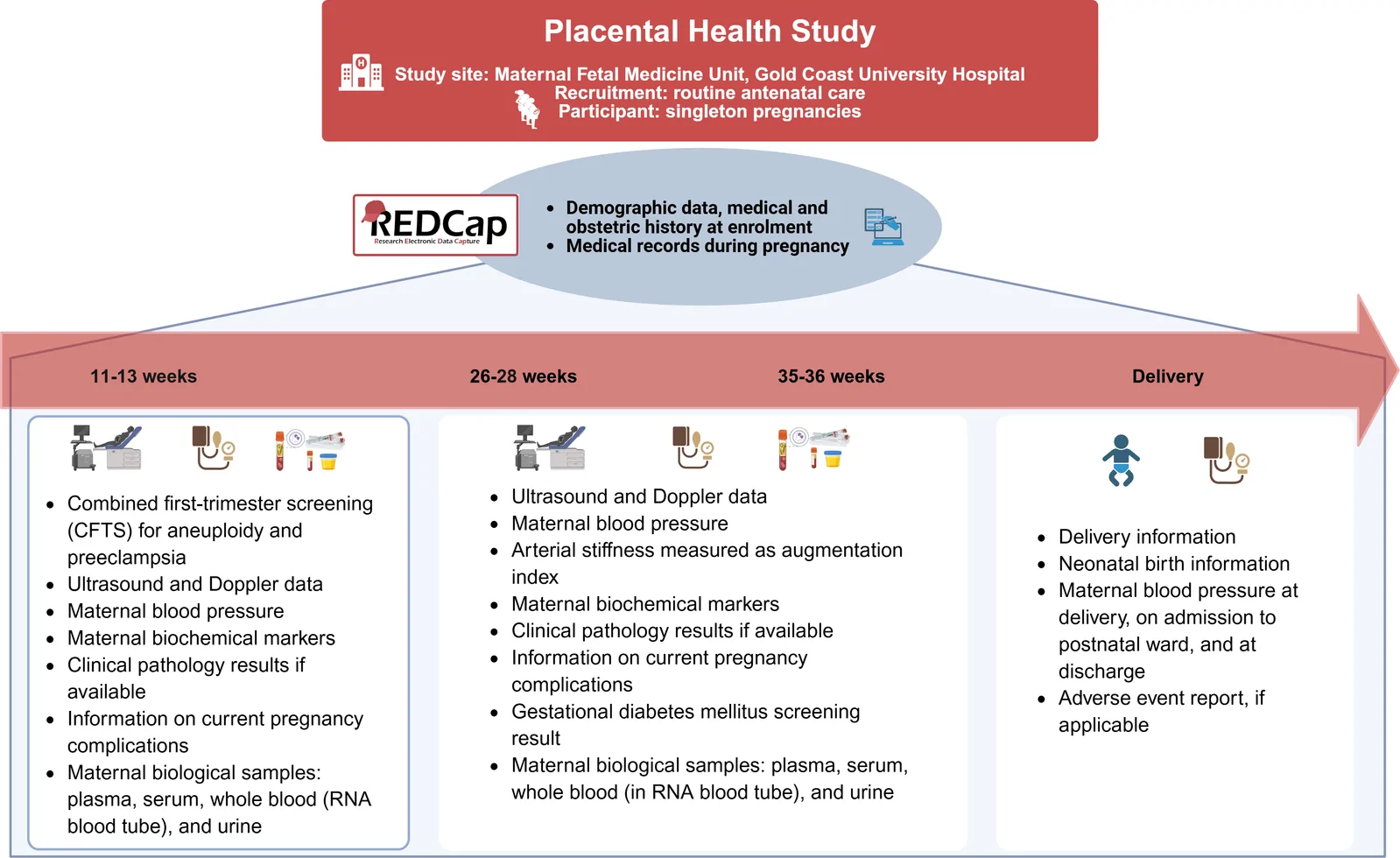
Overview of study design and data collection.

The Placental Health Study is a single-center, prospective cohort study conducted at the Maternal Fetal Medicine (MFM) Unit, Gold Coast University Hospital (GCUH), Queensland, Australia, and commenced in June 2022. Figure 1 provides an overview of the study design and data collection procedures.

### Study Objectives

#### Primary Objectives

The primary objectives of this study are as follows:

1. To improve current predictive models or develop new predictive models for placental dysfunction–related pregnancy complications, including preterm preeclampsia and FGR, at 2 critical gestational periods: the first trimester (11‐13 weeks) and the second trimester (26‐28 weeks)
2. To establish third-trimester (35‐36 weeks) models to predict term preeclampsia and FGR and to define the optimal time for delivery.

#### Secondary Objectives

The secondary objectives are as follows:

1. To predict the outcomes and prognosis of placental dysfunction–related complications by applying the developed predictive models
2. To evaluate the predictive value of clinically tested pregnancy biomarkers for pregnancy outcomes related to placental dysfunction
3. To investigate placental biological processes and their role in generating maternal circulating biomarkers associated with preeclampsia and FGR, thereby enhancing the understanding of uteroplacental dysfunction pathophysiology

### Study Population

Pregnant individuals presenting for clinical screening visits at the MFM unit, GCUH, are considered potentially eligible participants.

#### Inclusion Criteria

Participants are eligible for inclusion if they are aged ≥18 years, have a singleton pregnancy, have a gestational age <37 weeks at recruitment, and are able to read and provide written informed consent.

#### Exclusion Criteria

Participants are excluded if they have a multiple pregnancy; a pregnancy complicated by a major fetal anomaly, chromosomal abnormalities, or genetic syndromes; or preexisting maternal cardiac disease (including cardiomyopathy, heart failure, valvular disease, and ischemic heart disease).

### Ethical Considerations

#### Ethics Approval

This study received ethics approval from the Gold Coast Hospital and Health Service Human Research Ethics Committee (HREC/2022/QGC/84257) and the Griffith University Ethics Committee (2022/254). Any proposed amendments or substudies to the protocol will be reviewed and approved by the Study Steering Committee to ensure ongoing compliance with ethical standards.

#### Informed Consent

All participants provide written informed consent prior to enrollment. Participation is voluntary, and participants can withdraw at any time without impact on their current or future clinical care and may request the destruction of their previously collected data and samples.

#### Privacy and Confidentiality

All data and biospecimens are deidentified at collection using unique study IDs. Study data are collected and managed using REDCap (Vanderbilt University), a secure web-based platform hosted at Griffith University, in a reidentifiable format [30]. Biospecimens are stored in a reidentifiable format at –80 °C in the Pregnancy Research Laboratory at Griffith University. Access to study data and biospecimens is restricted to authorized research personnel only. All published data will be deidentified and presented in aggregate, ensuring participant confidentiality.

#### Compensation

No financial compensation is provided to study participants, as all study procedures are integrated into routine antenatal care and require no additional hospital visits. In addition, no costs are incurred by participants for study-related measurements.

### Recruitment and Retention

The recruitment strategy aligns with routine clinical visits at GCUH during 3 gestational periods of 11 to 13 weeks, 26 to 28 weeks, and 35 to 36 weeks. Women may join the cohort at any stage before 37 weeks of gestation. To facilitate the collection of longitudinal data and samples, participants enrolled in early to midgestation are encouraged to continue participation through delivery. Primary recruitment focuses on pregnant individuals attending the combined first-trimester screening at the MFM unit and includes referrals from all models of maternity care at Gold Coast Hospital and Health Service. Eligible women admitted to the GCUH maternity ward are also considered for inclusion. Clinical staff within the assessment units have been trained in the study protocol and eligibility criteria to ensure accurate information dissemination to potential participants. Eligible women expressing interest are approached by research midwives and clinic investigators for a detailed discussion of the study.

### Definitions of Preeclampsia and FGR

The diagnosis of preeclampsia follows the 2021 International Society for the Study of Hypertension in Pregnancy guideline [31]. Preeclampsia cases are subclassified based on gestational age at delivery as preterm preeclampsia (delivery <37 weeks) or term preeclampsia (delivery ≥37 weeks) [31]. FGR is defined in the absence of congenital anomalies according to international Delphi consensus criteria, with distinctions between early-onset and late-onset presentations (Textbox 1) [32].

Textbox 1. **Definitions of early- and late-onset fetal growth restriction [32].**

**Early-onset fetal growth restriction: gestational age <32 weeks**

- Abdominal circumference or estimated fetal weight <3rd percentile or umbilical artery absent end-diastolic flow

OR

- Abdominal circumference or estimated fetal weight <10th percentile *combined with*
- Uterine artery pulsatility index >95th percentile *and/or*
- Umbilical artery pulsatility index >95th percentile

**Late-onset fetal growth restriction: gestational age ≥32 weeks**

- Abdominal circumference or estimated fetal weight <3rd percentile

OR

At least 2 of the following 3:

- Abdominal circumference or estimated fetal weight <10th percentile
- Abdominal circumference or estimated fetal weight crossing >2 quartiles (50 percentiles) on growth charts (growth centiles are noncustomized)
- Cerebroplacental ratio <5th percentile or umbilical artery pulsatility index >95th percentile

### Data and Sample Collection

This study involves the collection of comprehensive maternal data, including demographics, medical and obstetric history, clinical assessments throughout pregnancy and delivery, and maternal biological samples.

#### Demographic and Clinical Data

Maternal demographic characteristics, medical and obstetric history are collected by research midwives, sonographers, and clinical research fellows at enrollment through face-to-face conversations or electronic medical records. Maternal risk factors are recorded in accordance with the National Institute for Health and Care Excellence (NICE) [33] and American College of Obstetricians and Gynecologists (ACOG) guidelines [34]. Clinical data, including routine ultrasound screenings, laboratory tests, and maternal and perinatal outcomes, are obtained from integrated electronic medical records.

#### Ultrasound and Biophysical Measurements

Ultrasound assessments, including fetal biometry, maternal and fetal Doppler ultrasound, and maternal ophthalmic artery Doppler, are performed by certified sonographers or MFM specialists in accordance with the International Society of Ultrasound in Obstetrics and Gynecology recommendations and guidelines [35-37]. Maternal cardiovascular function is evaluated through additional measurements, including blood pressure (BP) and arterial stiffness assessments. BP is measured using an Omron automated device (OMRON Healthcare Inc) following a standardized protocol, with MAP calculated as (2/3 diastolic BP + 1/3 systolic BP) based on the average of 2 stable readings [38]. Arterial stiffness, measured as augmentation index, is assessed using the Uscom BP+ device (USCOM Limited), according to the manufacturer’s guidelines.

#### Biological Sample Collection

All biological specimens are collected, processed, and stored according to standard operating procedures by trained research staff. At each visit, 15 mL of maternal blood and 5 mL of urine are collected. For serum collection, blood is collected in a nonanticoagulant vacutainer and allowed to coagulate at room temperature for 30 to 60 minutes, followed by centrifugation at 1300×*g* for 15 minutes at room temperature. Plasma samples are collected in an EDTA-treated vacutainer and immediately centrifuged at 1900×*g* for 15 minutes at room temperature. For RNA analysis, whole-blood samples are collected into PAXgene Blood RNA tubes, gently inverted 10 times for homogenization, stored upright at room temperature for 2 hours or longer, frozen at −20℃ for 24 hours, and stored at –80 ℃ for long-term preservation [39]. Random midstream urine samples are collected in sterile urine collection vials. Aliquots of serum (0.5 mL), plasma (0.5 mL), and urine samples (1 mL) are stored in 1.8 mL microtubes at –80 ℃.

#### Maternal Circulating Biomarker Measurements

Collected biological samples will be analyzed for maternal circulating biomarkers. Maternal serum levels of PlGF and sFlt-1 will be quantified using the KRYPTOR Compact Plus immunoanalyzer (BRAHMS Thermo Fisher Scientific), followed by calculation of the sFlt-1:PlGF ratio and multiples of the expected median using previously published equations [40,41]. In addition, exploratory omics analyses of maternal circulating biomarkers will be conducted using a nested case-control design on a subset of participants, using techniques such as quantitative polymerase chain reaction and liquid chromatography with quadrupole time-of-flight mass spectrometry.

### Statistical Analysis Plan

All statistical analyses will be conducted using the R statistical environment (R Foundation for Statistical Computing).

#### Sample Size Estimation

We estimate a 10% to 15% participation rate from the approximately 5000 annual deliveries at GCUH. On the basis of a prevalence of 10% for placental dysfunction–related disorders (preeclampsia and SGA or FGR), approximately 200 cases with these outcomes are expected. In the development of prediction models using binary outcomes (high-risk or low-risk) with a 10% incidence rate and up to 15 predictors, targeting an area under the receiver operating characteristic curve (AUC-ROC) of 0.75, the estimated sample size is approximately 1700 participants [42].

#### Descriptive Statistics

Continuous variables will be presented as either mean (SD) or median with IQR, depending on their distribution as assessed by visual inspection of histograms and normal quantile plots. Categorical variables will be described using frequencies and percentages. For comparisons between groups (pregnancies with and without placental insufficiency–related complications), independent-samples 2-tailed *t* tests or Mann-Whitney *U* tests will be used for continuous variables, and the chi-square test or Fisher exact test will be used for categorical variables, as appropriate.

#### Primary Objectives: Prediction Model Development

Multivariable logistic regression will serve as the primary analytical approach for prediction model development at 3 gestational windows (11‐13 weeks, 26‐28 weeks, and 35‐36 weeks of gestation) to address both primary objectives 1 and 2. Clinically relevant candidate predictors are predefined and entered into multivariable models, followed by model reduction to achieve parsimony (eg, backward elimination with a retention threshold of *P*<.10). Nonlinear relationships between continuous variables and the log odds of the outcomes will be tested with the inclusion of polynomial terms up to the third degree, which will be kept in the model if statistically significant.

For each patient, this method estimates a predicted probability of developing the outcome based on the log odds of the outcome. For primary objective 1, the predicted probabilities can be used to define low- and high-risk subgroups at predefined gestational age thresholds. Specifically, a risk threshold will be defined based on clinically relevant screen-positive rates (eg, 10%, 15%, or 20%) to balance sensitivity and specificity for clinical implementation. For primary objective 2, logistic regression will similarly provide predicted probabilities. In addition, Fine and Gray competing risks time-to-event models will be used as sensitivity analyses accounting for delivery without the outcome of interest (preeclampsia or FGR) as a competing event.

To further improve upon current FMF prediction models, a Bayesian updating approach will be applied to refine existing FMF first-trimester risk estimates using additional biomarkers and clinical variables collected later in pregnancy. The FMF first-trimester risk estimate will be used as the prior probability. Likelihood ratios derived from biomarker and biophysical measurements at 26 to 28 weeks and 35 to 36 weeks will be incorporated via Markov chain Monte Carlo simulations to generate updated posterior risk distributions. This enables dynamic, individualized risk reassessment as pregnancy progresses.

All models will undergo internal validation using 5-fold cross-validation with repeated iterations to obtain stable performance estimates. Model performance will be evaluated using the following metrics:

- Model discrimination: AUC-ROC and c-statistic
- Calibration assessment: calibration plots and calibration slope and intercept on cross-validated data
- Clinical utility: sensitivity, specificity, positive and negative predictive values, and screen-positive rates
- Comparison against existing risk assessment methods: NICE and ACOG guidelines for preeclampsia risk scoring

#### Secondary Objective Analysis

For secondary objective 1, time-to-event analyses will be used to evaluate the association between predicted risk scores and adverse maternal and perinatal outcomes after diagnosis of complications. For secondary objective 2, AUC-ROC analysis, sensitivity and specificity, and predictive values will be used to assess the individual predictive performance of each biomarker candidate. For secondary objective 3, longitudinal biomarker trajectories will be compared between participants with the outcomes of interest and those without these outcomes using linear mixed-effects models with random intercepts and slopes for each participant. Exploratory proteomics and metabolomics analyses will be performed on a subset of samples to identify novel biomarkers and biological pathways associated with placental dysfunction.

### Dissemination

The results of this study will be disseminated through publications in peer-reviewed scientific journals and conference presentations. We will codevelop a dissemination plan with Gold Coast Health and Hospital Service and consumers to communicate the project’s findings to key stakeholders (eg, consumers and policymakers). Additionally, participants have the option to receive a written summary of the study’s findings and future directions.

## Results

Recruitment for the Placental Health Study commenced in June 2022. By the close of recruitment in February 2026, a total of 1267 participants were enrolled. Of these, 1072 (84.6%) participants had a completed pregnancy, 144 (11.4%) participants are still to complete their pregnancy, and 51 (4%) had withdrawn or with missing or duplicated data. Comprehensive clinical data, including maternal demographics, medical and obstetric history, ultrasound measurements, Doppler indices, hemodynamic parameters, pathology laboratory results, and pregnancy outcomes, were collected through integration with electronic medical records (Table 1). Data were recorded in REDCap. Maternal serum, plasma, whole blood for RNA extraction, and urine samples have been collected and processed according to standardized operating procedures. Biospecimens are stored at −80 °C.

**Table 1.** Summary of data and sample collection throughout pregnancy.

Assessment period and category	Data collected
At enrollment	
Demographic data	Date of birth, age, height, weight, BMI, ethnicity, smoking, and alcohol use status
Medical history	Diabetes mellitus, chronic hypertension, systemic lupus erythematosus, and antiphospholipid syndrome
Obstetric history	Miscarriage, termination, parity, previous preterm birth, preeclampsia, FGR^a^, placental abruption, and placenta previa
Risk assessment	Preeclampsia risk scoring (NICE^b^ and ACOG^c^ guidelines)
11‐13 weeks assessment	
First-trimester risk assessment	NIPT^d^ results (risk of trisomies, fetal sex, risk of monosomy X, sex chromosome aneuploidy risk, 22q11.2 deletion risk, and fetal fraction [cfDNA^e^, %], FGR risk, and preterm preeclampsia risk
Ultrasound and Doppler	Fetal biometry (CRL^f^ and NT^g^), fetal Doppler (ductus venosus A-wave), maternal Doppler (right and left uterine artery PI^h^ and mean PI), biophysical markers (cervical length and measurement method), and ultrasound markers (nasal bone, tricuspid regurgitation, cardiac activity, FHR^i^, holoprosencephaly, diaphragmatic hernia, atrioventricular septal defect, exomphalos, and megacystis)
BP^j^	Systolic BP, diastolic BP, and MAP^k^
Biochemical markers	Free beta-hCG^l^, PAPP-A^m^, PlGF^n^, sFlt-1^o^, and sFlt-1:PlGF ratio
Clinical pathology	Urine test, full blood count, chemistry panel, and recent iron infusion date (if available)
26‐28 and 35‐36 weeks	
Ultrasound and Doppler	Fetal biometry (BPD^p^, HC^q^, AC^r^, FL^s^, EFW^t^, and DVP^u^); biophysical markers (cervical length and measurement method and placenta position); fetal Doppler (umbilical diastolic flow, UA^v^ PI Dopplers, MCA^w^ PI Dopplers, ductus venosus A-wave, and ductus venosus Dopplers-PIV^x^); and maternal Doppler (right and left uterine artery pulsatility index and mean PI)
Maternal hemodynamics	Systolic BP, diastolic BP, MAP, augmentation index, and maternal ophthalmic artery Doppler (right and left PI, RI^y^, EDV^z^, PSV^aa^-1, PSV-2, and PSV-2:PSV‐1)
Biochemical markers	PlGF, sFlt-1, and sFlt-1:PlGF ratio
Clinical pathology	Urine test, full blood count, chemistry panel, and recent iron infusion date (if available)
GDM screening	Random glucose, HbA_1c_^ab^, OGTT^ac^ (fasting, 1-hour, and 2-hour values); and insulin or metformin use
At delivery	
Delivery details	Gestational age at delivery; onset of labor; type of rupture of membrane; PPROM^ad^; FBS^ae^ test results; delivery type; number of vessels in cord; antenatal, intrapartum, and postnatal blood loss; postpartum hemorrhage
Neonatal outcomes	Live birth or stillbirth, onset of respirations, birth weight, birth length, head circumference, sex, Apgar scores (1 and 5 minutes), resuscitation, cord gas analysis, and SCN^af^ or NICU^ag^ admission
Maternal outcomes	BP, postpartum hemorrhage, and adverse events
All visits	
Pregnancy complication and medication	Preeclampsia, GH^ah^, antepartum hemorrhage, threatened preterm labor, FGR, PPROM, GDM^ai^; gestational age at diagnosis; medication details (start and stop date and dose)
Biological sample collection	Plasma, serum, whole blood (in PAXgene RNA blood tube), and urine

a FGR: fetal growth restriction.

b NICE: National Institute for Health and Care Excellence.

c ACOG: American College of Obstetricians and Gynecologists.

d NIPT: noninvasive prenatal testing.

e cfDNA: cell-free DNA.

f CRL: crown-rump length.

g NT: nuchal translucency.

h PI: pulsatility index.

i FHR: fetal heart rate.

j BP: blood pressure.

k MAP: mean arterial pressure.

l hCG: human chorionic gonadotropin.

m PAPP-A: pregnancy-associated plasma protein A.

n PlGF: placental growth factor.

o sFlt-1: soluble fms-like tyrosine kinase-1.

p BPD: biparietal diameter.

q HC: head circumference.

r AC: abdominal circumference.

s FL: femur length.

t EFW: estimated fetal weight.

u DVP: deepest vertical pocket.

v UA: umbilical artery.

w MCA: middle cerebral artery.

x PIV: pulsatility index for veins.

y RI: resistive index.

z EDV: end-diastolic velocity.

aa PSV: peak systolic velocity.

ab HbA_1c_: hemoglobin A_1c_.

ac OGTT: oral glucose tolerance test.

ad PPROM: preterm premature rupture of membranes.

ae FBS: fetal blood sampling.

af SCN: special care nursery.

ag NICU: neonatal intensive care unit.

ah GH: gestational hypertension.

ai GDM: gestational diabetes mellitus.

A subset of samples has undergone preliminary biomarker quantification, including measurement of angiogenic markers (sFlt-1 and PlGF), and additional omics-based analyses are underway. Interim analyses have been conducted comparing clinical variables and biomarkers between participants with and without complications. It is anticipated that 2 to 3 primary publications will be ready in late 2026 or early 2027, including evaluation of maternal circulating biomarkers and exploratory analyses of novel biomarker candidates identified through omics techniques.

## Discussion

This protocol describes a prospective cohort study aimed at developing advanced prediction models for placental dysfunction–related pregnancy complications, allowing for dynamic and personalized accurate risk assessment throughout the pregnancy. First- and second-trimester prediction models aim to enhance early risk assessment for placental dysfunction–related complications (preeclampsia, FGR, preterm birth, and stillbirth), and the third-trimester model focuses on predicting the onset of term complications (preeclampsia and FGR) and determining optimal delivery timing. This comprehensive strategy advances personalized obstetric care through evidence-based risk stratification.

The FMF first-trimester prediction model shows superior performance compared with traditional risk-factor–based checklists [17]. By combining maternal factors with measurements of MAP, UtAPI, and serum PlGF, this model is the most effective method for predicting early-onset and preterm preeclampsia, achieving detection rates of 90% and 75%, respectively [22,43]. This model has been validated across diverse populations, consistently demonstrating predictive performance comparable to the original studies and has been tested in randomized clinical trials [24,44-48]. Importantly, when coupled with prophylactic low-dose aspirin administration starting before 16 weeks’ gestation in high-risk pregnancies, this screening strategy reduces preterm preeclampsia by more than 60% [24,49]. In addition, the model achieves detection rates of 56% for early SGA fetuses (<32 weeks) and 46% for preterm SGA neonates (<10th percentile), with aspirin use potentially reducing the incidence of preterm SGA by 20% and early SGA by 40% [23].

These advancements in preterm preeclampsia screening have prompted updates to national [50,51] and international [52] guidelines, recommending implementation of the prediction model with prophylactic low-dose aspirin to reduce preterm preeclampsia incidence and associated adverse outcomes. A cost-effectiveness analysis in Australia demonstrated significant benefits of this screening strategy, including prevention of 31 preterm preeclampsia cases and cost savings of approximately Aus $1.43 million (Aus $1=US $0.72 as of August 27, 2020) over a 2-year period [53]. Despite these clinical benefits, the FMF first-trimester prediction model has not yet become a standard component of routine maternity care for all health care providers in Australia. Gold Coast University Hospital has implemented FMF-based first-trimester preeclampsia screening in routine clinical care, providing a valuable setting to generate evidence supporting broader application in clinical practice.

Current prediction models still have limited accuracy for predicting term preeclampsia and SGA due to their complex etiology. Although considered less severe, term complications constitute most cases, occurring at least twice as frequently as preterm preeclampsia, and impose a significant health care burden [26]. While the first-trimester screening combined with low-dose aspirin effectively prevents preterm preeclampsia, it showed limited efficacy for term preeclampsia [24]. A prediction model at 35 to 36  weeks’ gestation, using maternal factors and biomarkers, can identify approximately 70% of pregnancies at risk of developing term preeclampsia [54]. In addition, planned birth at term is a potential prevention strategy for term preeclampsia [55,56]. A recent secondary analysis from a prospective nonintervention cohort study has demonstrated that a risk-stratified approach based on a combined prediction model at 35 to 36  weeks’ gestation with timed birth is likely able to reduce the risk of term preeclampsia [27]. However, research on individualized risk assessment models at late gestation to determine optimal delivery timing remains limited. This cohort study will facilitate the development of refined prediction models and optimal delivery timing strategies to reduce term preeclampsia incidence and improve pregnancy outcomes.

This project aims to enhance existing prediction models for term preeclampsia and SGA through the evaluation of additional biomarkers across 3 critical gestational stages, investigating both routine antenatal measurements and specialized markers of placental and cardiovascular dysfunction, including Doppler ultrasound parameters, maternal hemodynamic indices, and angiogenic markers. Doppler ultrasound assessment of uterine arteries can reflect placental perfusion status, with elevated values indicating increased resistance and impaired placental blood flow [57,58]. Maternal hemodynamic parameters, including ophthalmic artery Doppler indices, MAP, and augmentation index, provide insights into cardiovascular adaptation and peripheral vascular function [59-61]. Angiogenic markers (sFlt-1, PlGF, and the sFlt-1:PlGF ratio) indicating angiogenic imbalances serve as biochemical predictors of placental dysfunction [20,62,63] and spontaneous preterm labor [64]. Furthermore, our study establishes a valuable biobank for identifying novel molecular biomarkers and investigating underlying mechanisms of placental dysfunction through omics techniques. Incorporating these diverse markers and novel biomarker candidates into existing prediction models has the potential to enhance accuracy for late-onset pregnancy complications.

The key strength of this prospective cohort study lies in its comprehensive approach to implementing and improving the prediction of placental dysfunction–related complications throughout pregnancy, from first-trimester screening to late-gestation risk stratification in a general-risk population. The proposed dynamic prediction framework, if validated, could benefit implementation of targeted surveillance protocols and timely interventions, addressing current gaps in evidence-based obstetric care. Furthermore, the study establishes a valuable database and perinatal biorepository, providing valuable resources for novel biomarker discovery and investigation of associations between clinical phenotypes and biological mechanisms underlying maternal systemic changes and placental dysfunction. Some limitations should be considered. First, this study is limited to singleton pregnancies, which may restrict its applicability to multiple pregnancies. Second, the single-center design potentially constrains the generalizability of the findings to other populations and clinical settings. Third, despite incorporating diverse clinical, biochemical, and biophysical markers, some important predictors may remain unmeasured. In addition, some biomarkers in the final prediction models may be expensive or technically challenging to measure in routine clinical practice, potentially limiting widespread adoption. External validation in independent populations is planned as a future phase of research and will evaluate model transportability and generalizability across different clinical settings and populations. Despite the limitations, this research holds significant potential to advance our understanding of placental dysfunction–related pregnancy complications and enhance obstetric care through improved risk prediction and personalized intervention strategies.

## Supplementary material

10.2196/95231 Peer Review Report 1 Peer review report by the Gold Coast Health Collaborative Research Grant Scheme

10.2196/95231 Peer Review Report 2 Grant agency's outcome report

## Abbreviations

ACOG: American College of Obstetricians and Gynecologists
AUC-ROC: area under the receiver operating characteristic curve
BP: blood pressure
FGR: fetal growth restriction
FMF: Fetal Medicine Foundation
GCUH: Gold Coast University Hospital
MAP: mean arterial pressure
MFM: Maternal Fetal Medicine
NICE: National Institute for Health and Care Excellence
PlGF: placental growth factor
sFlt-1: soluble fms-like tyrosine kinase-1
SGA: small for gestational age

## References

[1] Wardinger JE, Vadakekut ES (2025). StatPearls [Internet].

[2] Hoffman MK (2023). The great obstetrical syndromes and the placenta. BJOG.

[3] Dimitriadis E, Rolnik DL, Zhou W (2023). Pre-eclampsia. Nat Rev Dis Primers.

[4] Damhuis SE, Ganzevoort W, Gordijn SJ (2021). Abnormal fetal growth: small for gestational age, fetal growth restriction, large for gestational age: definitions and epidemiology. Obstet Gynecol Clin North Am.

[5] Say L, Chou D, Gemmill A (2014). Global causes of maternal death: a WHO systematic analysis. Lancet Glob Health.

[6] Triggs T, Crawford K, Hong J, Clifton V, Kumar S (2024). The influence of birthweight on mortality and severe neonatal morbidity in late preterm and term infants: an Australian cohort study. Lancet Reg Health West Pac.

[7] Fox A, McHugh S, Browne J (2017). Estimating the cost of preeclampsia in the healthcare system: cross-sectional study using data from SCOPE study (screening for pregnancy end points). Hypertension.

[8] Fox H, Callander EJ (2020). The cost of Hypertensive Disorders of Pregnancy to the Australian healthcare system. Pregnancy Hypertens.

[9] Marzouk A, Filipovic-Pierucci A, Baud O (2017). Prenatal and post-natal cost of small for gestational age infants: a national study. BMC Health Serv Res.

[10] Pittara T, Vyrides A, Lamnisos D, Giannakou K (2021). Pre-eclampsia and long-term health outcomes for mother and infant: an umbrella review. BJOG.

[11] Sławek-Szmyt S, Kawka-Paciorkowska K, Ciepłucha A, Lesiak M, Ropacka-Lesiak M (2022). Preeclampsia and fetal growth restriction as risk factors of future maternal cardiovascular disease-a review. J Clin Med.

[12] Takahashi M, Makino S, Oguma K (2021). Fetal growth restriction as the initial finding of preeclampsia is a clinical predictor of maternal and neonatal prognoses: a single-center retrospective study. BMC Pregnancy Childbirth.

[13] Audette MC, Kingdom JC (2018). Screening for fetal growth restriction and placental insufficiency. Semin Fetal Neonatal Med.

[14] American College of Obstetricians and Gynecologists’ Committee on Practice Bulletins—Obstetrics and the Society for Maternal-Fetal Medicine. (2019). ACOG Practice Bulletin No. 204: Fetal Growth Restriction. Obstet Gynecol.

[15] Staff AC (2019). The two-stage placental model of preeclampsia: an update. J Reprod Immunol.

[16] Burton GJ, Jauniaux E (2018). Pathophysiology of placental-derived fetal growth restriction. Am J Obstet Gynecol.

[17] Tan MY, Wright D, Syngelaki A (2018). Comparison of diagnostic accuracy of early screening for pre‐eclampsia by NICE guidelines and a method combining maternal factors and biomarkers: results of SPREE. Ultrasound Obstet Gynecol.

[18] Chen JY, Yu BL, Wu XJ, Li YF, Zhong LY, Chen M (2024). A longitudinal and cross-sectional study of placental circulation between normal and placental insufficiency pregnancies. Placenta.

[19] Cao L, He B, Zhou Y, Chen T, Gao Y, Yao B (2024). Utility of uterine artery Doppler ultrasound imaging in predicting preeclampsia during pregnancy: a meta-analysis. Med Ultrason.

[20] Stepan H, Galindo A, Hund M (2023). Clinical utility of sFlt-1 and PlGF in screening, prediction, diagnosis and monitoring of pre-eclampsia and fetal growth restriction. Ultrasound Obstet Gynecol.

[21] Cuffe JS, Holland O, Salomon C, Rice GE, Perkins AV (2017). Review: placental derived biomarkers of pregnancy disorders. Placenta.

[22] Tan MY, Syngelaki A, Poon LC (2018). Screening for pre‐eclampsia by maternal factors and biomarkers at 11–13 weeks’ gestation. Ultrasound Obstet Gynecol.

[23] Tan MY, Poon LC, Rolnik DL (2018). Prediction and prevention of small-for-gestational-age neonates: evidence from SPREE and ASPRE. Ultrasound Obstet Gynecol.

[24] Rolnik DL, Wright D, Poon LC (2017). Aspirin versus placebo in pregnancies at high risk for preterm preeclampsia. N Engl J Med.

[25] Wikström AK, Larsson A, Eriksson UJ, Nash P, Nordén-Lindeberg S, Olovsson M (2007). Placental growth factor and soluble FMS-like tyrosine kinase-1 in early-onset and late-onset preeclampsia. Obstet Gynecol.

[26] von Dadelszen P, Syngelaki A, Akolekar R, Magee LA, Nicolaides KH (2023). Preterm and term pre-eclampsia: relative burdens of maternal and perinatal complications. BJOG.

[27] Magee LA, Wright D, Syngelaki A (2023). Preeclampsia prevention by timed birth at term. Hypertension.

[28] Foo FL, Mahendru AA, Masini G (2018). Association between prepregnancy cardiovascular function and subsequent preeclampsia or fetal growth restriction. Hypertension.

[29] Reddy M, Palmer K, Rolnik DL, Wallace EM, Mol BW, Da Silva Costa F (2022). Role of placental, fetal and maternal cardiovascular markers in predicting adverse outcome in women with suspected or confirmed pre-eclampsia. Ultrasound Obstet Gynecol.

[30] Harris PA, Taylor R, Minor BL (2019). The REDCap consortium: building an international community of software platform partners. J Biomed Inform.

[31] Magee LA, Brown MA, Hall DR (2022). The 2021 International Society for the Study of Hypertension in Pregnancy classification, diagnosis & management recommendations for international practice. Pregnancy Hypertens.

[32] Gordijn SJ, Beune IM, Thilaganathan B (2016). Consensus definition of fetal growth restriction: a Delphi procedure. Ultrasound Obstet Gynecol.

[33] (2019). Hypertension in pregnancy: diagnosis and management. National Institute for Health and Care Excellence.

[34] (2020). Gestational hypertension and preeclampsia: ACOG practice bulletin, number 222. Obstet Gynecol.

[35] Salomon LJ, Alfirevic Z, Da Silva Costa F (2019). ISUOG practice guidelines: ultrasound assessment of fetal biometry and growth. Ultrasound Obstet Gynecol.

[36] Sotiriadis A, Hernandez-Andrade E, da Silva Costa F (2019). ISUOG practice guidelines: role of ultrasound in screening for and follow-up of pre-eclampsia. Ultrasound Obstet Gynecol.

[37] Kane SC, Brennecke SP, da Silva Costa F (2017). Ophthalmic artery Doppler analysis: a window into the cerebrovasculature of women with pre-eclampsia. Ultrasound Obstet Gynecol.

[38] Roberts L, Chaemsaithong P, Sahota DS, Nicolaides KH, Poon LC (2017). Protocol for measurement of mean arterial pressure at 10-40 weeks’ gestation. Pregnancy Hypertens.

[39] PAXgene blood RNA tubes (IVD). QIAGEN.

[40] Nucci M, Poon LC, Demirdjian G, Darbouret B, Nicolaides KH (2014). Maternal serum placental growth factor (PlGF) isoforms 1 and 2 at 11-13 weeks’ gestation in normal and pathological pregnancies. Fetal Diagn Ther.

[41] Zeisler H, Llurba E, Chantraine F (2016). Predictive value of the sFlt-1:PlGF ratio in women with suspected preeclampsia. N Engl J Med.

[42] Riley RD, Ensor J, Snell KI (2020). Calculating the sample size required for developing a clinical prediction model. BMJ.

[43] Chaemsaithong P, Sahota DS, Poon LC (2022). First trimester preeclampsia screening and prediction. Am J Obstet Gynecol.

[44] Lobo GA, Nowak PM, Panigassi AP (2019). Validation of Fetal Medicine Foundation algorithm for prediction of pre-eclampsia in the first trimester in an unselected Brazilian population. J Matern Fetal Neonatal Med.

[45] Guizani M, Valsamis J, Dutemeyer V (2018). First-trimester combined multimarker prospective study for the detection of pregnancies at a high risk of developing preeclampsia using the Fetal Medicine Foundation-algorithm. Fetal Diagn Ther.

[46] Park FJ, Leung CH, Poon LC, Williams PF, Rothwell SJ, Hyett JA (2013). Clinical evaluation of a first trimester algorithm predicting the risk of hypertensive disease of pregnancy. Aust N Z J Obstet Gynaecol.

[47] Oliveira N, Magder LS, Blitzer MG, Baschat AA (2014). First-trimester prediction of pre-eclampsia: external validity of algorithms in a prospectively enrolled cohort. Ultrasound Obstet Gynecol.

[48] Chaemsaithong P, Pooh RK, Zheng M (2019). Prospective evaluation of screening performance of first-trimester prediction models for preterm preeclampsia in an Asian population. Am J Obstet Gynecol.

[49] Rolnik DL, Wright D, Poon LC (2017). ASPRE trial: performance of screening for preterm pre-eclampsia. Ultrasound Obstet Gynecol.

[50] (2023). Hypertension in pregnancy guideline. Society of Obstetric Medicine Australia and New Zealand.

[51] (2024). Early pregnancy screening and prevention of preterm preeclampsia and related complications (C-Obs 61). RANZCOG.

[52] Poon LC, Shennan A, Hyett JA (2019). The International Federation of Gynecology and Obstetrics (FIGO) initiative on pre‐eclampsia: a pragmatic guide for first‐trimester screening and prevention. Int J Gynaecol Obstet.

[53] Park F, Deeming S, Bennett N, Hyett J (2021). Cost-effectiveness analysis of a model of first-trimester prediction and prevention of preterm pre-eclampsia compared with usual care. Ultrasound Obstet Gynecol.

[54] Panaitescu A, Ciobanu A, Syngelaki A, Wright A, Wright D, Nicolaides KH (2018). Screening for pre-eclampsia at 35-37 weeks’ gestation. Ultrasound Obstet Gynecol.

[55] Koopmans CM, Bijlenga D, Groen H (2009). Induction of labour versus expectant monitoring for gestational hypertension or mild pre-eclampsia after 36 weeks’ gestation (HYPITAT): a multicentre, open-label randomised controlled trial. Lancet.

[56] Grobman WA, Rice MM, Reddy UM (2018). Labor induction versus expectant management in low-risk nulliparous women. N Engl J Med.

[57] Liu Y, Xie Z, Huang Y, Lu X, Yin F (2024). Uterine arteries pulsatility index by Doppler ultrasound in the prediction of preeclampsia: an updated systematic review and meta-analysis. Arch Gynecol Obstet.

[58] Khong SL, Kane SC, Brennecke SP, da Silva Costa F (2015). First-trimester uterine artery Doppler analysis in the prediction of later pregnancy complications. Dis Markers.

[59] Gurgel Alves JA, Praciano de Sousa PC, Bezerra Maia E Holanda Moura S, Kane SC, da Silva Costa F (2014). First-trimester maternal ophthalmic artery Doppler analysis for prediction of pre-eclampsia. Ultrasound Obstet Gynecol.

[60] Franz MB, Burgmann M, Neubauer A (2013). Augmentation index and pulse wave velocity in normotensive and pre-eclamptic pregnancies. Acta Obstet Gynecol Scand.

[61] Zhu J, Zhang J, Syaza Razali N, Chern B, Tan KH (2021). Mean arterial pressure for predicting preeclampsia in Asian women: a longitudinal cohort study. BMJ Open.

[62] Tomimatsu T, Mimura K, Matsuzaki S, Endo M, Kumasawa K, Kimura T (2019). Preeclampsia: maternal systemic vascular disorder caused by generalized endothelial dysfunction due to placental antiangiogenic factors. Int J Mol Sci.

[63] Stepan H, Herraiz I, Schlembach D (2015). Implementation of the sFlt-1/PlGF ratio for prediction and diagnosis of pre-eclampsia in singleton pregnancy: implications for clinical practice. Ultrasound Obstet Gynecol.

[64] Chaiworapongsa T, Romero R, Tarca A (2009). A subset of patients destined to develop spontaneous preterm labor has an abnormal angiogenic/anti-angiogenic profile in maternal plasma: evidence in support of pathophysiologic heterogeneity of preterm labor derived from a longitudinal study. J Matern Fetal Neonatal Med.

