# Supplementary material for: Prediction of Pregnancy Complications Related to Placental Dysfunction: Protocol for a Prospective Cohort Study (Placental Health Study)
Source: JMIR Res Protoc. 2026 Aug 25;15:e95231. doi: 10.2196/95231 (PMC13504624; doi:10.2196/95231)
Supplement: Supplementary file 1 [file resprot-v15-e95231-s001.pdf]

Gold Coast Health Collaborative Research Grant Scheme

Gold Coast Health Queensland Government | Gold Coast Hospital Foundation | Griffith University | Bond University | Southern Cross University

Enquiries to: Office Research Governance and Development
Telephone: 07 5687 0663
Email: grantsgoldcoast@health.qld.gov.au

Date: 08 November 2021

Dear Fabricio

On behalf of the Research Grants Scheme Working Party, I am pleased to inform you that your grant application has been approved for funding in the 2021 round of the Gold Coast Health Collaborative Research Grant Scheme.

Application ID:	RGS2021-036 – Prof Fabricio da Silva Costa
Project Title:	Predicting placental dysfunction outcomes with biochemical, cardiovascular and ultrasound markers
To the amount of:	**$92,557** (to be confirmed)

The Office for Research Governance and Development will send an invitation for your project team to attend the Research Showcase on the 29th November 2021 at 9.30am until 12.00pm to receive the award for your grant. During this presentation you are asked to present an overview of your research during a 5-minute presentation. Please arrive by 9.00am so the order of proceedings can be explained to you. A PowerPoint template will be provided to you for this purpose. Until public announcement on 29th November, the outcomes of this grant scheme remain under **embargo**. You may share the outcome with your research team only.

In review of the proposed budget against the study protocol, it was identified that additional information is required before your budget can be finalised. You will need to arrange a meeting with the Chair to discuss the details of your budget and make any suggested revisions. You will also receive a meeting invitation in due course to discuss the grant details and establishment of a Funding Agreement between yourself as the Lead Principal Investigator and the funding partners. We also ask that you give some consideration to milestones for the duration of your project for discussion at the meeting.

Congratulations, we wish you every success in your research.

Yours Sincerely

Andrea Marshall

Prof Andrea Marshall
Chair, Research Grant Scheme Working Party
Gold Coast Health and Hospital Service
Gold Coast University Hospital
1, Hospital Boulevard, Southport, Qld 4215

RGS2021-036

Application ID	RGS2021-036

Assessment Forms

RGS 2021 - Full Application Assessment Scientific

Project Title Long	Predicting placental dysfunction outcomes with biochemical, cardiovascular and ultrasound markers
Comments - Significance	The investigators have provided a detailed review of the relevant literature in this field which identifies important health outcomes related to placental insufficiency that potentially affect a significant (5-10%) proportion of the pregnant population.<br>The investigators have identified prospective markers that could be used in a multivariate algorithm.<br>The study has the potential to improve our knowledge and understanding of these individual markers ands their interactions.<br>They could have added further evidence of potential benefit of reducing false positive rates associated with current suggested methods of screening.<br>Positive findings would likely change clinical practice.
Scientific quality - Comments	There are four clearly stated research questions which align with the literature review.<br>The methodology appears appropriate for the aims of the study although it was not completely clear how the sample size was calculated.<br>The investigators identify a prevalence of pre-eclampsia of 5-8% and state that they want to recruit 263 women who develop this condition: If the prevalence is 5% this would require a population of 5260. The suggested sample size (1800) should produce:<br>90 women who develop pre-eclampsia (at min 5% prevalence)<br>54 women who develop FGR (at min 3% prevalence)<br>126 women who deliver <37 weeks (at 7% prevalence).<br>From experience, these numbers of affected pregnancies should be enough to determine screening efficacy of individual and combinations of markers - and this population should be accessible at GCH - but it would be good to see a better description of power / sample size before starting the study.<br>It is not clear to me that the fourth aim (long term maternal cardiovascular state) is achievable within the timeline for this study.
Budget - Comments	The request for support is limited to human resource (0.4FTE midwife for two years) and as such this is a well defined budget.<br>I note that the investigators have already secured another source of funding for the lab work and have developed university and commercial links to manage other costs.<br>The project will require human resource for management and I can see that this is a critical issue to facilitate success.

Overarching Comments	Please check the sample size calculation and identify the power for recognizing differences in efficacy between different screening tools.
**REVIEWER 2**	
Comments - Significance	I believe the background provide including review of literature, identification of gaps and justification of approach are well presented.
Scientific quality - Comments	I believe all aspects of the study methods were covered well, except more detail could have been provide in the data analysis section, aligning the different approaches of analysis to meet the study objectives....a range of analysis being undertaken but no explanation of how this links to forming an algorithm and how the analysis answers the objectives being asked. A degree of confidence that this will lead to the outcomes stated comes from the study synopsis indicating the outcomes achieved from the pilot data. Ideally this section in your protocol could be strengthened.
Budget - Comments	This budget is unfortunately lacking detail in relation to in-kind costs associated with investigators involved, sample collection, sonography requirements. There are many components to a budget and even for the in-kind or no cost components, it is important to clarify and justify how these aspects are resourced, to ensure confidence that the project outcomes will be achieved.
Overarching Comments	The project has high significance and there is a clear justification for approach being taken. I would consider expanding on the data analysis section considerably to provide the reviewer more understanding of how the different types of analysis are linked to the outcomes being measured. Also how the algorithm will be developed?<br>The budget is lacking considerable detail given the amount of data collection being undertaken. Detailing in-kind and no cost is still helpful to understand that all aspects for completing the project have been accounted for and therefore the project is well resourced. With the information provided, this was difficult to know. Although there is some information in different parts of the protocol that helps explain some of the resource costs, a well detailed budget would certainly have assisted your application.<br>One other aspect to note is where the data is being stored. The assumption is a QLD Health computer but no detail provided. More detail would be required here for an ethics application.

RGS 2021- Full Application Assessment Non-Scientific

Comments - Significance	The purpose of the scheme is to facilitate:<br>1) A strong research culture within GCH facilities; 2) increased research capacity of GCH and its staff; 3) meaningful collaborations between GCH research active staff and our academic partners; 4) translatable research that addresses one or more of the core challenges that are significant to delivering healthcare to our communities; and 5) high-quality health and health system related research that has impact locally, nationally and internationally.<br><br>Based on content written in this section, this grant aligns with 3 of the

	purposes above (1, 3, 5). Authors have done a great job articulating this section, well done.
Comments – research capability and capacity building	Excellent research capability<br>Good capacity building- wanted to know a little more about what the sonographers/HDR candidates would do and a bit more explicit details about what "exposure" entailed explicitly for the other staff
Comments - Collaboration and consumer engagement	Excellent collaboration.<br>Excellent consumer engagement
Comments - Impact	Clearly defined impacts.<br>Plans for how these may translate to measurable effects on the health care for Gold Coast was not explicit.
Overarching Comments	Main strength: Collaborative team and great consumer engagement<br>Main limitation: more explicit plans for research capacity building would strengthen
**REVIEWER 2**	
Comments - Significance	This is an important piece of work that can potentially help to improve outcomes for women and their babies. Building on the scientific knowledge and how this will support clinical care is clear.
Comments – research capability and capacity building	The team has the requisite expertise to undertake this study and has considered how this research will help build research capacity across a range of clinicians working in fetal maternal medicine. More detail on the specific plans for capacity building would have been helpful, such as specific learning outcomes or goals for those being mentored would have strengthened this section.
Comments - Collaboration and consumer engagement	The institutional collaboration is strong and will benefit GCH. I can see that there is some links to consumer engagement but it is unclear how consumers will directly influence this particular study. While this work is aligned with the Stillbirth CRE, this is a separated from GCH so it would be helpful to know how the consumers in this group are going to work with researchers on this study, what role they will play and whether they have helped to inform the original study protocol.
Comments - Impact	The impact statement suggests promise for developing predictive algorithms. It isn't clear from this statement whether this study alone will generate sufficient data for this work or if additional data are required. Informing future world-class research is important and would have a positive impact on GCH. Being more explicit on whether the data from this study alone would help with translation to practice would have been helpful.
Overarching Comments	As above.

RGS 2021 - Full Application Assessment - Spokesperson

Comments - Significance	This application presents a clear background that makes a case for the study proposed.This is an area of significant clinical importance.. There is the opportunity to create an outcome that would have local, national and potentially international relevance. The study outcomes would underpin future work presumably testing the model & algorithms

	developed. The method chosen seem appropriate & this does feel a solid study.
Comments – research capability and capacity building	I felt this was the weakest aspect of this application. the team is obviously well led by leading clinical researchers I this field but the description of the capacity building is not strongly embedded in the proposal. the project is led by senior researchers with no sense of how the ECRs and MCRs will be mentored and supported or how the new clinical researchers will be developed and with what skills.
Comments - Collaboration and consumer engagement	Again I found this element of the project underwhelming. Whilst the strength and benefits of this collaboration are obvious, they are not well described. It is not clear how other than parallel recruitment how the organisations will work together on and across the project. consumer involvement appears weak and tokenisitic. There is no indication they will be part of any study advisory group, or support the development of study materials for example<br><br>A point of note on budget - the proposal suggests that the ELIZA tests will be funded by the grant but I do not see them included in the budget
Comments - Impact	There is a plan included for dissemination and it does involve consumer input. The aims of this study align explicitly with GCH vision and purpose to improve outcomes - this study also has the ability to extend beyond the reach of GCH. there is a articulation of the intention to underpin future Cat 1 grant applications with this work.
Overarching Comments	This study addresses an area of clinical significance & is well designed to answer the research aims. It undoubted has the ability to inform local practice & beyond.<br><br>I felt the weakest elements were in the descriptions of<br>1. How the ECR & MCRs woukd be mentored & supported<br>2. How the collaborators would work in & across the project<br>3. A failure to really embed consumer engagement - or at least to articulate that clearly<br><br>I was also slightly confused - re budget - the proposal suggests that the ELIZA tests will be funded by the grant but I do not see them included in the budget
