# Supplementary material for: Prediction of Pregnancy Complications Related to Placental Dysfunction: Protocol for a Prospective Cohort Study (Placental Health Study)
Source: JMIR Res Protoc. 2026 Aug 25;15:e95231. doi: 10.2196/95231 (PMC13504624; doi:10.2196/95231)
Supplement: Supplementary file 2 [file resprot-v15-e95231-s002.pdf]

2021 HEALTH GROUP SEED GRANTS
APPLICATION FORM

Please Note: Researcher (CI-A) and some Project details will have been collected via the online application form.

RESEARCH TEAM

You are required to attach ALL team members CVs to the online application form. Max. 2 page CV (see Health Research Hub for suggested content).

**CHIEF INVESTIGATOR (B)**	
Name	Fabricio da Silva Costa
Title	Professor
Contact email address	fabricio.dasilvacosta@health.qld.gov.au
**CHIEF INVESTIGATOR (C)**	
Name	Karl Kristensen
Title	Associate Professor
Contact email address	karl.kristensen@health.qld.gov.au
**CHIEF INVESTIGATOR (D)**	
Name	Anthony Perkins
Title	Professor
Contact email address	a.perkins@griffith.edu.au
**CHIEF INVESTIGATOR (E)**	
Name	Olivia Holland
Title	Research Fellow
Contact email address	o.holland@griffith.edu.au
**CHIEF INVESTIGATOR (F)**	
Name	Kirsten Palmer
Title	Senior Research Fellow
Contact email address	kirsten.palmer@monash.edu
**CHIEF INVESTIGATOR (G)**	
Name	Daniel Rolnik
Title	Senior Research Lecturer
Contact email address	daniel.rolnik@monash.edu
**CHIEF INVESTIGATOR (H)**	
Name	
Title	
Contact email address	

PROJECT OR INITIATIVE DESCRIPTION *maximum 1 page, font size 11 point and essential references only. Include (where appropriate): Aims and specific hypothesis, background and short-term measurable outcomes*

Aims: To create algorithms using angiogenic markers, markers of endothelial dysfunction, placental ageing, and maternal cardiovascular indices to better predict 1) the onset of pre-eclampsia (PE)/placental insufficiency, 2) the outcomes of PE/placental insufficiency, 3) the optimal timing of birth and 4) long-term cardiovascular morbidity in women after a pregnancy affected by PE/placental insufficiency.

Background: PE is a heterogeneous disease, which affects 2-8% of pregnancies and is a leading cause of maternal and fetal morbidity and mortality. Despite extensive research, we are still unable to predict and prevent the outcomes of PE, and perhaps this is the reason why there has been little change in outcomes over the last decade. Predictive models and therapeutic agents have largely targeted individual aspects of pre-eclamptic pathophysiology and have failed to take account of the heterogeneous nature of this condition. This has hampered our ability to prevent and treat this disease. Our research aims to create a model that can better predict the onset, outcomes, and long-term sequelae of PE and, as a secondary aim due to its large association, to predict fetal growth restriction (FGR). We hope to achieve this by accounting for the heterogeneous nature of pre-eclamptic pathophysiology using a combination of cardiovascular, angiogenic and endothelial markers in our prediction model. Furthermore, we will explore whether these abnormalities persist beyond the puerperium, and whether this confers a higher risk of long-term cardiovascular disease. Our pilot data in 126 high-risk women with suspected or confirmed PE showed the combination of mean arterial pressure, uterine artery pulsatility index, augmentation index and ophthalmic artery peak ratio was able to predict composite maternal and perinatal adverse outcomes and necessity of birth before 34 weeks with an AUROC of 0.70 (95% CI 0.61, 0.79) and 0.84 (95% CI 0.76, 0.93), respectively. Now, we propose to test these algorithms + blood biomarkers in a general obstetric population.

Short term measurable outcomes: There are three **Primary Outcomes**:

1. Development of pre-eclampsia and other pregnancy complications related with placental insufficiency
2. Identification between 24-28 weeks gestation of a population with low and high risk of placental insufficiency
3. Identification at 36 weeks of signs of placental ageing that indicates necessity of early planned birth

We expect to show that by combining markers of endothelial function and maternal cardiovascular indices at 24-28 weeks gestation, we can create a reliable clinical model that can predict a high-risk group, and outcomes of PE and other pregnancy complications related to placental insufficiency. At 36 weeks, we expect these markers could also predict the best time for birth. We also expect that these biochemical and cardiovascular abnormalities will persist in a proportion of women that developed placental insufficiency, conferring a higher risk of long-term cardiovascular morbidity.

Significance (innovation, transformative potential & alignment to research strengths): This research will be of benefit to all women with disorders of pregnancy involving placental insufficiency. The results may alter our clinical processes and affect the way we predict, monitor, and change the outcomes of placental insufficiency. It may also influence future therapies targeted at the prevention and management of placental insufficiency. As well as optimising pregnancy outcomes, this research also has the potential to predict and prevent stillbirth. As such, this aligns very well with the work of the Stillbirth Centre of Research Excellence (Stillbirth CRE) of which CIA is a co-Director. The launch of the Stillbirth National Action & Implementation Plan at the end of last year indicates that stillbirth prevention is a national health priority. The national stillbirth prevention strategy (the Safer Baby Bundle) includes better identification and management of fetal growth restriction, as well as optimising timing of birth, and so there is considerable crossover with this proposed study. This study will also create a partnership of two leading Australian clinical and research Maternal-Fetal Medicine (MFM) Units, establishing a collaboration between the Gold Coast University Hospital (GCUH) MFM Unit and the Monash Medical Centre (MMC) MFM Unit, and with Griffith University's Pregnancy Research Group. These three clinical and research centres have substantial published expertise in clinical and basic science research related to PE and placental insufficiency and the initial results of the current study in a general obstetric population will help to substantiate data already collected in symptomatic high blood pressure patients. These data will help to attract funding from nationally competitive grants schemes, such as the NHMRC and MRFF.

This study will be conducted at the MFM Unit, Gold Coast University Hospital (GCUH) and at the MFM Unit, Monash Medical Centre (MMC). The primary source of patient recruitment will be the Women's Outpatient clinic at GCUH and the Maternal Assessment Centre and Antenatal Clinics at MMC. Additional sources will include pregnant women from community antenatal care on the Gold Coast and Southeast Melbourne. The laboratory experiments will be conduct at the Pregnancy Research Lab (Griffith University) coordinated by CID Perkins.

METHODOLOGY *(maximum 1.5 pages)*

Methodological Approach

This study will be conducted as an observational prospective cohort study. As described above, the aims of this study are to determine whether certain pathophysiological aspects of PE/placental insufficiency can be used to predict 1) disease onset and outcomes and 2) long term sequelae. We believe that a prospective cohort study is the ideal way to address these aims as it allows us to follow a specific cohort of women in a non-invasive manner and observe specific pathophysiological changes in a longitudinal manner in the second half of pregnancy. The aims of this study are not amenable to a randomised control trial as no intervention is required.

Study population

Women with a singleton pregnancy before 28 weeks gestation will be approached for recruitment. Overall, we hope to recruit approximately 1000 participants over a period of 12 months, and as PE/placental insufficiency would develop in around 5-8% of this cohort there should be between 50 to 80 women with these outcomes. Laboratory experiments will be completed along with the data collection and final outcomes and project results will be available within 18 months. PE will be defined in accordance with the 2014 International Society for the study of Hypertension in Pregnancy (ISSHP) guidelines. Small for gestational age (SGA): fetuses with estimated fetal weight (EFW) and/or abdominal circumference (AC) below the 10th, but above the 3rd centiles, with normal maternal and fetal Dopplers. Fetal growth restriction (FGR): EFW and/or AC below the 3rd centile or EFW and/or AC below the 10th centile with abnormal Dopplers. Preterm birth: spontaneous or elective delivery before 37 weeks gestation.

Inclusion criteria

Pregnant women before 28 weeks gestation.

Exclusion criteria

Multiple pregnancy, pregnancy complicated by major fetal anomaly or genetic syndrome, known underlying maternal cardiac disease (cardiomyopathy, heart failure, valvular disease, ischaemic heart disease), age less than 18, women incapable of giving valid consent (diminished understanding, non-English speaking, and interpreter unavailable), participation in a study where there is a pharmaceutical intervention.

Data collection

Following recruitment, women will complete a questionnaire of their demographics, including maternal age, ethnicity, body mass index, medical and obstetric history, and current pregnancy complications. We will then measure various indices to assess cardiovascular, endothelial, placental, and fetal function. This will involve measurement of blood pressure, blood and urine tests, a fetal ultrasound, and a maternal cardiovascular ultrasound.

Specific indices measured

1) Biochemical markers of PE (routine) – these will include urinary protein:creatinine ratio, full blood count, liver function tests and urea/electrolytes/creatinine. These investigations are routinely performed in pregnancies complicated by gestational hypertension and PE, generally on a weekly basis. They will therefore be processed as part of routine clinical care through the GCUH and Monash Pathology Departments.

2) Angiogenic markers, markers of endothelial dysfunction and mRNAs- These will be assessed using enzyme-linked immunosorbent assay (ELISA) and will include sFlt-1, sFlt-1 e15a, PlGF, ICAM-1 and VCAM-1. This will involve obtaining an additional 20mL of blood from the participants. These investigations will be performed at the Pregnancy Research Laboratory, Griffith University. sFlt-1/PlGF analysis will be performed with in-kind support from ThermoFisher, the ELISA's and mRNAs analysis required will be funded by the grants obtained for this study.

3) Markers of cardiovascular function – Maternal cardiovascular function will be assessed by the following:

a) Uterine artery Doppler, which will be measured bilaterally and sampled 1cm downstream from where the uterine artery crosses the external iliac artery. The pulsatility index and other relevant waveform characteristics will be recorded.

b) Ophthalmic artery Doppler will be measured by pulse wave Doppler interrogation to generate a time-velocity dicrotic waveform. From this, the pulsatility index, resistance index and peak ratio will be calculated. Studies have shown that cerebrovascular haemodynamics can be reflected directly through assessment of the ophthalmic artery Doppler.

c) Mean arterial pressure will be measured using Omron automated blood pressure cuffs.

d) Arterial stiffness will be measured using a USCOM BP+ device, which can be used to measure central and peripheral blood pressures and the arterial stiffness augmentation index.

4) Markers of fetal wellbeing – All fetal scans will be performed by an accredited primary investigator involved in this research at 24-28 weeks and at 36 weeks. Accreditation to perform growth ultrasounds will be obtained through GCUH and Monash MFM Units. Fetal markers will include fetal growth (estimated fetal weight, abdominal circumference, femur length) and Doppler indices (umbilical artery, middle cerebral artery, and ductus venosus).

5) Placenta – Placental tissue will be obtained from women who have provided consent to this aspect of the project. Tissue will be obtained during both normal deliveries and caesarean sections according to the protocols set out by the Global Pregnancy Collaboration (CoLab) and currently in use through the QLD family Cohort. Samples will be collected as quickly as possible after delivery and ideally within the first ten minutes.

The clinical and laboratory-based CIs have extensive expertise in all techniques described above.

Frequency of review and investigations

Most indices will be assessed at the time of recruitment at 24-28 and 36 weeks and 6 months postpartum. Fetal growth scans will be performed at the time of recruitment at 24-28 weeks and at 36 weeks. It is important to note that all patients will routinely perform OGTT at 24-28 weeks and cases of non-proteinuric gestational hypertension and PE have regular blood and urine tests as a part of standard clinical monitoring. Our tests will be performed in parallel. Therefore, most blood tests will not incur extra intervention to patients – the additional tests will be analysed from an extra 20mL blood collected at the time of routine monitoring.

ROLE OF PERSONNEL *(Explain the role of the Cis and any other personnel including ECRs, maximum 0.5 page)*

CIA Ellwood, CIB Costa, and CIF Palmer worked together on the conception and design of the study. CIC Kristensen and CIG Rolnik will assist with data collection together with one PhD student from each institution. CIG Rolnik will perform data analysis; CID Perkins and CIE Holland will provide laboratory analysis. All CIs will participate in the interpretation of the findings, drafting the related articles, critical revision of the articles and final approval of the versions to be published.

Both CIA Ellwood and CIB Costa are well established senior clinical researchers with extensive track records of research in adverse pregnancy outcomes which is relevant to this study, as well as being clinically involved in the delivery of the ultrasound services required. CID Perkins and CIE Holland are involved in internationally recognised placental biochemistry researched and are ideally placed to provide the laboratory support required. CIC Kristensen and CIF Palmer are early to mid-career clinical researchers whose track records are developing and will both be involved in future submissions for national-competitive grants. As MFM clinicians they are integral to the success of the project. The project will also allow two health professionals (senior sonographers - one at each site), to enrol as HDR candidates thus building capacity for future research activity.

TIMELINE, MILESTONES AND BUDGET

Detail the timeline, milestones and budget for this project at six monthly intervals. For projects longer than 6 months, you will need to provide six monthly reports in relation to these milestones.

Timeline	Milestone	Tasks	Resources Required	Budget breakdown	TOTAL *(input data from this column into the online form)*
PART 1<br>*0 – 6 months*	Initiation (Start-up)	Acquisition of blood and placenta collection material<br>Acquisition of BP+ and blood pressure devices	Blood collection material<br>Placental collection consumables<br>Blood pressure and augmentation index devices, centrifuge	Procedure gloves ($348)<br>Needles and holders ($7,042)<br>Placental collection ($12,000)<br>Centrifuge ($2,500)<br>BP+ ($3,937), Omrom BP ($590)<br>Tubes: blood tubes (gold, purple, RNA) and cryotubes ($9,420)	*$35,857*
PART 2<br>*6-12 months*	Data collection and lab analysis	Blood and placenta collection and lab assays	General lab reagents<br>Western blot reagents<br>Enzyme assays<br>Biomarker ELISA assays<br>Placental biomarkers	General lab reagents ($4,500)<br>Western blot reagents ($1,000)<br>Enzyme assays ($6,500)<br>Biomarker ELISA assays ($15,000)<br>Placental biomarkers ($6,600)	*$33,600*

PART 3<br>*12-18 months*	Finalise lab analysis<br>Outcome data collection and data analysis<br>External funding applications and paper production	Blood and placenta lab analysis<br>Ultrasound and clinical data analysis	None	None	*$0*
				TOTAL REQUESTED	*$69,457*

FUNDING

1.	Describe leverage funding available for this study e.g. Industry partner funding, in kind support, research co-ordination support, PhD scholarships, other grants that will contribute to your study.

- Ultrasound equipment is already available at the GCUH and MMC MFM Units and these machines are accessible for this study. Laboratory equipment will be provided by the Pregnancy Research Laboratory at Griffith University coordinated by CID Perkins. All the required infrastructure and expertise for the biochemical analysis is in place.
- ThermoFisher will support sFlt-1/PlGF analysis with in-kind provision of a Kryptor analyser and kits for 2000 tests (investment over $100k).
- Griffith Pregnancy Research Lab in-kind support for -80C freezer.
- Research coordination for CIB Costa is provided by the GCUH MFM Unit throughout with 10 hours of protected research time per week.
- CIE Holland currently holds a Fellowship from the Lions Medical Research Foundation.
- CID Perkins currently has grant support from the Norman Beischer Medical Research Foundation and the Allen Foundation (US) on related projects using metabolomics to diagnose placental pathologies.

2.	Explain how this funding will support you to obtain external competitive funds for your research – What is the proposed timeline for external grant submission?

The funding will help our group to quickly start this study and to generate pilot data in the next 12 months in a general obstetric population. This will be added to our pilot collected data in a high-risk population to support future competitive grant applications such as NHMRC and MRFF. Applications for either Ideas grants, or other targeted funding rounds will be prepared for submission in 2022. This funding will help our group to create predictive algorithms for PE / placental insufficiency in a general risk obstetric population. The algorithms will be prospectively validated in a near future multicentre study that would be very competitive to attract funding support and that could be rapidly translated in clinical practice, potentially improving maternal and perinatal outcomes in our community and across Australia and save health care funds.

HEAD OF ELEMENT APPROVAL

Please outline your support of this project and its alignment to area/s of research strengths in your element	This project provides a good foundation for a strong future research collaboration between the School of Medicine & Dentistry, GCUH MFM Unit, the Griffith Pregnancy Research Group and with Monash University. As CIA is also Head of Element this has been discussed with the DHOS (Research).
Signature	[signatures]
Date	30 April 2021

SUBMISSION DETAILS

Combine your 2-page CV and the Research Teams CVs into **ONE** pdf document called **Your Surname_2022_CVs** to upload to the online form.

Save application - **Your Surname_2022_HTH SEED GRANT** and upload to the online form.

Online form available on the **HEALTH RESEARCH HUB**
